# Feature generation and contribution comparison for electronic fraud detection

**DOI:** 10.1038/s41598-022-22130-2

**Published:** 2022-10-27

**Authors:** Yen-Wu Ti, Yu-Yen Hsin, Tian-Shyr Dai, Ming-Chuan Huang, Liang-Chih Liu

**Affiliations:** 1 College of Artificial Intelligence, Yango University, Fuzhou, Fujian 350001 China; 2 Institute of Finance, National Yang Ming Chiao Tung University, Hsinchu, 30010 Taiwan; 3 Department of Information Management and Finance and Institute of Finance, National Yang Ming Chiao Tung University, Hsinchu, 30010 Taiwan; 4 Institute of Computer Science and Engineering, National Yang Ming Chiao Tung University, Hsinchu, 30010 Taiwan; 5 Department of Information and Finance Management, National Taipei University of Technology, Taipei, 10608 Taiwan

**Keywords:** Computer science, Information technology

## Abstract

Modern money transfer services are convenient, attracting fraudulent actors to run scams in which victims are deceived into transferring funds to fraudulent accounts. Machine learning models are broadly applied due to the poor fraud detection performance of traditional rule-based approaches. Learning directly from raw transaction data is impractical due to its high-dimensional nature; most studies construct features instead by extracting patterns from raw transaction data. Past literature categorizes these features into recency, frequency, monetary, and anomaly detection features. We use various machine learning algorithms to examine the performance of features in these four categories with real transaction data; we compare them with the performance of our feature generation guideline based on the statistical perspectives and characteristics of (non)-fraudulent accounts. The results show that except for the monetary category, other feature categories used in the literature perform poorly regardless of which machine learning algorithm is used; anomaly detection features perform the worst. We find that even statistical features generated based on financial knowledge yield limited performance on a real transaction dataset. Our atypical detection characteristic of normal accounts improves the ability to distinguish them from fraudulent accounts and hence improves the overall detection results, outperforming other existent methods.

## Introduction

According to statistics from the Communications Fraud Control Association, in 2019 alone, fraud caused a global loss of 28.3 billion U.S. dollars;^1^ the funds that law enforcement agencies are able to recover in such cases are extremely limited. Many commercial banks adopt a rule of thumb to develop automated detection systems for electronic fraud in which they form a set of rules or filters to spot real fraudulent accounts from a lengthy list of suspicious activities created based on past observations or suggestions from police agencies. However, a Taiwan bank working with our research team reports that this rule-based method yields a poor recall rate (5.56%) and precision rate (40%).

To address these problems, many studies have begun to use machine learning to detect electronic funds transfer (EFT) fraud. However, training a machine learning model with raw transaction data is impractical due to its high-dimensional nature^2^. Past studies find that feature engineering is essential to improving the performance of most machine learning models^3–6^; that is, they extract patterns such as criminal behaviors from raw data to construct features. We attempt to collect extracted features that can be applied to our transaction datasets from past research^3–5,7–11^ and compare their detection performance. According to the definition proposed by Baesen et al.^11^ these features can be categorized into recency, frequency, monetary (RFM), and anomaly features. These features are almost all based on statistical characteristics and do not involve financial expertise. However, past research and our own experiments have shown that these features alone do not fully describe users’ historical transaction records, nor do they reflect the relationship between legal transactions and fraud crime^7^. Hence, we construct reasonable features inspired by the anti-money laundry (AML) guidelines from Financial Supervisory Commission, R.O.C. and related financial know-how and observations. These features can be divided into basic statistical features and features for identifying (non)-fraudulent accounts. The first part includes transaction frequencies, (less-)frequently used transaction types, phone/internet transaction usage, and the trends and standard deviations of deposit/withdrawal amounts. These features are selected by taking AML guidelines into account by monitoring accounts with frequent transactions and frequent access to automatic/online services. The second type of features captures patterns of fraudulent accounts with reference to financial knowledge and cases provided by judicial authorities. For example, fraudsters often repeatedly withdraw cash using the ATM’s Fast Cash function, causing their last withdrawal amount to be greater than the remaining account balance. Third, we also capture the patterns of normal accounts that are seldom involved in fraudulent acts, such as counter services. The quality of our proposed features can be verified using the feature importance ranking criteria proposed by Butaru et al.^12^ published in a premium financial journal. Our eight proposed features’ importance scores rank high and occupy two of the top five.

To further confirm the effect of our feature engineering, we use a real transaction dataset to compare the performance of all the features mentioned above in detecting EFT fraud in different machine learning models. Of RFM features and anomaly detection features, only monetary features have F1 scores exceeding 50%. Other categories of RFM features and anomaly detection features perform poorly in our experiments. In addition to the above features, we also evaluate features based on the financial profession proposed by Xie et al.^7^ The experimental results show that our unique features for detecting normal accounts improve the ability to distinguish them from fraudulent accounts. Our proposed features yield the best performance when applied to the extreme gradient boosting (XGBoost) model compared to all other features and models mentioned above.

However, for financial institutions such as banks, the machine learning model used must be explainable to ensure (1) that there are no unpredictable results and (2) that there are no illegal practices or other unfairness^13^. Using our proposed features with the XGBoost model provides superior performance and excellent interpretability. As mentioned earlier, we draw from professional financial knowledge to yield novel and effective features. Based on a real transaction dataset, we compare the features proposed in the literature with those proposed here to construct a practical and efficient model by which to detect EFT fraud and provide a reference for banks to improve their operating procedures.

We organize the rest of the paper as follows. In  "Related work" section, we review related work on the detection of financial frauds. In "Methods" section, we introduce our methods of feature engineering. Section "Feature construction" describes how we perform feature engineering, "Features based on atypical detection techniques" section describes the features we generate based on our observations, and "Features for comparison" section describes the feature categories in the literature. Section "Experiments" describes the training procedure and compares the fraud detection performance among different machine learning models. Section "Conclusion and future research" concludes.

## Related work

Many studies have been conducted on financial fraud detection, especially regarding credit card fraud cases^2,10,14,15^. This is in addition to research on the detection of fraud cases such as phone fraud^16^, online transaction fraud^17^, and instant payment service fraud^18^. Traditionally, to meet the requirements of laws and regulations, banks have used interpretive rule-based anti-fraud models. However, such models are easy to crack: as long as the fraudsters know the rules, they can avoid behavior described by the rules. Therefore, over the years, researchers have begun to turn to complex models. Cheng et al.^2^ aggregates features such as the current transaction amount, the average transaction amount, the total amount, the transaction time, and the most recent transaction location to detect credit card fraud. The attention mechanism is used to extract important features about time and space such as the most recent transactions, several recent transactions, or the transactions that have occurred recently in a given city. The above features are sent to a 3D CNN with max pooling, followed by a fully connected layer and an output layer to predict whether the transaction is fraudulent or legitimate. Noting that there is far more unlabeled data than labeled data in the real world, Wang et al.^18^ address this problem for instant payment fraud detection. They use user-interaction information, user-nick graphs, as well as information about frequently-used locations from online merchants to build a heterogeneous attribute graph, and then apply semi-supervised graph embedding to the graph to obtain a low-dimensional representation for each node. To produce interpretable detection results, they apply attention to each node to calculate the relations of different neighbors or different attributes, and then apply the attention again to correlate different data views. Since it is difficult to obtain complete cross-bank EFT data, Zheng et al.^16^ use two datasets from two banks in China to detect suspicious transfers. They use a GAN with a denoising autoencoder to calculate the probability that a transfer is fraudulent in the receiving bank. Similar to our approach, the features they extracted from the datasets include basic information about the transfer. outsourced fraud detection service providers for legitimate reasons. Xie et al.^7^ pointed out that only through effective feature engineering can an effective fraud detection model be created. But prevailing feature constructions based on transaction frequency is far from perfect since it is difficult to distinguish between fraud and normal transaction behavior. Therefore, they proposed some features for detecting credit card fraud based on financial knowledge and we have adopted a similar approach. Zheng et al.^16^ use denoising autoencoders to extract fraudulent features to improve detection. They report a high recall rate (about 80%) but a low precision rate (about 4%).

Many machine learning models are black boxes, that is, we do not know how the models make their decisions. This is however unacceptable in the fields of finance, law, and medicine. According to indicators proposed by Moraffah et al. in 2020^13^, our proposed model is causally interpretable, that is, it explains the results of prediction and classification.

The features we propose meet the needs of banks.

## Methods

### Feature construction

In this work, we do not use public datasets in our experiments, as the limited nature of the information provided in such datasets makes them unsuitable for verifying the proposed model. The source of our data is the transaction data of a Taiwanese bank (denoted as Bank T) from April 2018 to September 2018 and the account watch list from the National Police Agency of the Ministry of the Interior. This dataset allows us to recognize the transactions of all (non)-fraudulent accounts in Bank T. Each transaction consists of the de-identified account ID, the transaction type (e.g., inter-bank transferral, deposit, etc.); the transaction date; the withdrawal (or deposit) amount; the balance; and the “note” feature, which contains data such as textual information like “transferred to company X” or the ATM ID representing the machine ID if the transaction was performed through an ATM.

We select the last several raw transaction data from each account as aggregated data for generating features that effectively characterize the account. We find that including the last nine transactions strikes a good balance between losing (fraudulent) accounts and retaining (fraud) transactions for training. Specifically, aggregating more transactions prevents accounts with fewer transactions from training, whereas aggregating fewer transactions causes the feature generation procedure to involve fewer transaction data. Including nine transactions for each account maximizes the number of transactions involved in the feature generation process. By selecting the last nine transactions for each account as aggregated data, 373869 transactions are involved in feature generation; 372375 (1494) features were extracted from non-fraudulent (fraudulent) accounts.

To get the most from this limited data, we created new financial features from the aggregated data using methods similar to those proposed by Whitrow et al.^5^ and Bhattacharyya et al.^3^ The feature engineering process we use here is called feature construction (generation)^19^, the purpose of which is to better understand the defining features of fraudulent and non-fraudulent behavior. Most current feature engineering results are based on transaction frequency, which has clear shortcomings. A lack of financial expertise makes it difficult to distinguish fraud from normal withdrawal behavior, and captures only the time features of user withdrawal records. Most current research on fraud detection uses machine learning models to directly process raw data, which results in similar sets of features^4,11^. User behavior patterns, however, are not easily characterized by such features. For example, a client’s past behavior patterns may not reflect the behavior brought about by changes in his/her life. A client may change occupations or get married, which could lead to changed withdrawal patterns.

On the other hand, fraudsters may behave like normal users to fool the system. Hence, features based on time and frequency alone are unreliable. Therefore, we manually construct atypical features based on professional financial knowledge and cases provided by law enforcement agencies.

Specifically, we use or generate three categories of features for each account: (1) statistics-based features from the last nine transactions, (2) features that closely resemble those of normal accounts, and (3) features that closely resemble those of fraudulent accounts w.r.t. phone fraud. After creating these features, we train state-of-the-art machine learning models to learn the relationship between the created features and (non)-fraudulent accounts, in particular eXtreme gradient boosting (XGBoost), Bayes point machines, decision forests, support vector machines (SVM), neural networks (NN), decision jungles, and logistic regression.

First, based on the aggregated data, we list the statistics of the selected features and use them as basic features according to each account’s behavior as shown in Table 1.

**Table 1:** Basic statistical features of nine most recent transactions for each account.

Basic statistical features		Features that characterize fraudulent accounts	
Feature	Description	Feature	Description
*BankID*	The bank the account belongs to	*MFTAC*	Number of times this account transferred to its most frequently ransferred account
*AVG_T_Interval*	(Date of ninth transaction − date of first transaction)/9	*Cash_Withdrawal_Limits*	Number of debit transactions with amount of 10005, 20005, or 60000 TWD
*24_hr_Transaction*	Transactions made in last 24 hours	*Withdrawal_After_Deposit*	Number of withdrawals right after deposits right after deposit transferred account
*Most_Frequent_Act*	Most frequent type of transaction	*LDT>R*	Whether the account’s last withdrawal is more than its remaining balance
*Second_Most_Frequent_Act*	Second most frequent type of transaction	*Weighted_Fraud_Coefficient*	(LDT>R)$\times$(Number_ATM$+0.1$)/9 $\times$(Cash_Withdrawal_Limits$+0.1$)/9 $\times$(Withdrawal_After_Deposit$+0.1$)/9
*Least_Frequent_Act*	Least frequent type of transaction	*Suspicious_Branch*	Whether account makes transactions in areas that are dense in fraudulent activitics
*Note_Not_NE*	Number of non-empty values in Note column	*HFW_Bank_of_ATM*	Whether account is opened at a bank in which fraudsters often open accounts
*Voice*	Number of times Voice service was used		
*Internet*	Number of times E-bank service was used		
*Credit_STDev*	Standard deviation of withdrawal amount		
*Debit_STDev*	Standard deviation of deposit amount		
*Savings_Gradient*	Gradient of trend of remaining balance		
*Number_ATM*	Number of ATM transactions		
**Features characterizing non-fraudulent accounts**			
*LT_Signature*	Number of transaction types conducted only by legal accounts		
*LATM_Signature*	Number of ATMs used by fewer than six fraudulent accounts		

Second, to generate features that highlight the properties of legitimate accounts, we compare the transaction types of legitimate accounts and fraudulent accounts and group these into two categories: transaction types adopted only by fraudulent (legitimate) accounts, none of which are used by legitimate (fraudulent) accounts. Similarly, for those ATMs which are used by at least (fewer than) six fraudulent accounts, we consider them as frequently (rarely) used for fraud. Hence, when an ATM rarely used for fraud is used by an account, it is viewed as an ATM conducting what is likely to be a normal transaction. These features concerning legitimate transactions and ATMs are shown as part of Table 1.

Last, we observe the modus operandi in phone fraud, and attempt to derive representative features. For example, fraudsters are likely to simultaneously withdraw money from an account through different ATMs multiple times. Given these observations, we propose several features that characterize the modus operandi, as shown in Table 1. Further, we jointly consider the simultaneous events of (1) the withdrawal limit of each ATM, (2) the withdrawal occurring immediately after a deposit, and (3) a remaining balance that is less than the previous withdrawal amounts, and calculate the *Weighted_Fraud_Coefficient* in Table 1. Furthermore, in the proposed model we also use as features (1) whether each withdrawal from an account is always the maximum amount set by the bank, (2) whether the daily withdrawal amount from an account is always the maximum amount set by the bank, and (3) the locations where fraud activities occur frequently. We also use these features to build other novel features that yield good results, as described in the next section.

### Features based on atypical detection techniques

Our novel approach takes into account personal behavior patterns and regionality, and includes features that are not used in other similar studies. We list these features below.

**Characteristic 1.** Savings gradient

Fraudsters have a tendency to withdraw as much and as frequently as possible, as the victim’s account is added to the watch list soon after the fraudsters’ behaviors are discovered.

That is, fraudulent acts are often characterized by a large negative slope of the linear regression model fitting an account’s remaining balance.

**Characteristic 2.** Legal account

Some transaction types are rarely executed by fraudsters, such as making frequent small withdrawals at ATMs over a long period of time. If an account never has such transactions, then it is relatively likely that this account is involved in fraud.

We define this feature as *LT_Signature*.

**Characteristic 3.** Last withdrawal

This feature is called *LDT*>*R*. To quickly withdraw a deposit from an account, fraudsters often use the ATM’s fast cash function, which can cause the last withdrawal amount to exceed the deposit amount.

**Characteristic 4.** Withdrawal_After_Deposit

This is the *Withdrawal_After_Deposit* feature. To quickly withdraw a deposit from an account, fraudsters often make a withdrawal from the account immediately after the victim deposits the money.

**Characteristic 5.** Feature integration

To reduce the false alarm rate, we observe several suspicious features at the same time and use their product to evaluate the possibility of a transaction being fraudulent. The features used for reference include *LDT*>*R* and *Withdrawal_After_Deposit*. We also observe whether the withdrawal amount is the same as the limit set by the bank (i.e., *Cash_Withdrawal_Limits*) and the number of times the account holder uses the ATM (i.e., *Number_ATM*). The equation we use to calculate the value of the feature is defined as

1 $$ \begin{gathered}   (LDT > R) \hfill \\    \times (Number\_ATM + \alpha )/n \hfill \\    \times (Cash\_Withdrawal\_Limits + \alpha )/n \hfill \\    \times (Withdrawal\_After\_Deposit + \alpha )/n, \hfill \\  \end{gathered}  $$

where *n* is the number of observed transactions for the account and $\alpha$ is a small number greater than 0, which is added to avoid a final value of 0 just because the value of one of the above features is 0.

In our experiments, we set $\alpha$ to 0.1 and *n* to 9, which means that we observe nine transactions for each account. We define this feature as *Weighted_Fraud_Coefficient*.

**Characteristic 6.** Specific account

This feature is called *MFTAC*. Fraudsters sometimes have their own money laundering channels that allow them to make frequent transfers to a number of fixed accounts.

**Characteristic 7.** ATM

This feature is called *LATM_Signature*. Different environments in various places mean that fraud cases also exhibit regional characteristics. Some ATMs are more frequently used by fraudsters.

**Characteristic 8.** Feature integration

This feature is called *HFW_Bank_of_ATM*. Each bank has its own rules for opening accounts and using ATMs; thus fraudsters prefer to use accounts from specific banks.

The statistical significance of atypical detection techniques can be measured by the feature importance of the features generated by the technique, as will be discussed in Table 3. We also conducted a leave-one-out feature selection experiment ^20^ to show that removing any of the above features generally reduces the detection performance of the machine learning model significantly, as shown in Table 4.

### Features for comparison

In this article, we further classify the features we use. In addition to observing the performance of machine learning models, we also attempt to understand the effects of different types of features used in various machine learning models.

Recency, frequency, and monetary (RFM) analysis is an important part of feature engineering which is used widely by academia and industry. Therefore, we first discuss features created by other researchers based on RFM analysis; some of the features applicable to our model are listed in Table 2. Frequency-related features are mainly related to the time elapsed since the last occurrence of an event that satisfies the features’ conditions/descriptions in the right column of Table 2. Those features that Baesens et al.^11^ classify as frequency-related pertain mainly to the number of transactions that meet certain conditions during a predetermined period of time. Such features are usually transactions that have been aggregated in the last period of time in the dataset. The last item in the RFM analysis is features related to the transaction amount, which are classified as monetary features by Baesens et al.^11^ This type of feature is mainly based on tracking the transaction amount within a certain period and related parameters such as the average and standard deviation.

In addition to RFM features, Baesens et al.^11^ also propose a feature classification called features based on (unsupervised) anomaly detection techniques (FBADT). This type of feature is also based on statistical data but does not directly use the value of the target variable; rather, it observes the data pattern and detects samples that differ from most data patterns. In Table 2 we list the RFM and FBADT features used in the literature that are applicable to our real transaction dataset.

**Table 2:** RFM and FBADT features from the literature that are applicable to our dataset.

Feature Categories	Feature description
Recency	Transactions made in the last 24 hours
Recency	The average of the sum of deposits and withdrawals in the past 30 days
Recency	The average of the daily sum of the deposit and withdrawal amounts in the past 30 days
Recency	The average daily transaction amount for a specific account within 30 days.
Recency	The average weekly remittance amount in the past 60 days
Recency	The average weekly transaction amount for a specific account within 60 days.
Frequency	Assume that the suspicious behavior with the most occurrences of the account in nine days is S. This feature counts the occurrences of S.
Frequency	(Date of the ninth transaction minus date of the first transaction)/9.
Frequency	Least frequent type of transaction.
Frequency	Most frequent type of transaction.
Frequency	Second most frequent type of transaction
Monetary	Standard deviation of the withdrawal amount.
Monetary	Standard deviation of the deposit amount.
Monetary	Is the withdrawal amount the same as the withdrawal amount that fraudsters are accustomed to?
Monetary	The total transaction amount
Monetary	The total amount of consecutive transaction types being conducted.
Monetary	The median absolute deviation of the transaction amount of each account.
Monetary	The average number of occurrences of the number “0” in the transaction amount.
Monetary	Maximum daily transaction amount of the account.
Monetary	The maximum sum of the number of withdrawals and deposits of the account on the same day.
Monetary	The number of times the withdrawal amount of an account is greater than the maximum transaction amount minus the maximum fast transfer amount.
Monetary	The number of times the withdrawals of an account in a single day are greater than the maximum transaction amount minus the maximum fast transfer amount.
Monetary	The number of times that the withdrawal amount of an account is lower than the minimum fast transfer amount.
Monetary	The number of times the transaction amount of an account is greater than 95% of all transaction amounts.
Monetary	The number of times that an account has withdrawn all its balances.
FBADT	Whether the Mahalanobis distance of an account’s features exceeds the threshold set by the bank
FBADT	The average distance between the feature vector of an account and the feature vectors of the five closest accounts.
FBADT	The average density of the neighborhood of the five closest accounts divided by the average density of the neighborhood of the account.
FBADT	Take the group of isolation trees with the highest degree of isolation of observation to calculate the average standard path length of all tree
FBADT	Whether the first digit of the transaction amount satisfies the distribution of Newcomb-Benford law
FBADT	The number of account transactions in which the Z-score of the amount is greater than 3

We collect as many of the features proposed in the literature as possible, find those features suitable for our real transaction dataset to conduct experiments, and observe the effects when using these four types of features to detect EFT fraud in our dataset. A comparison of the results with the proposed features based on the feature engineering of the financial profession confirms that the proposed features and rules in Section "Features based on atypical detection techniques" improve the performance for detecting financial crime, especially when using the XGBoost model, as illustrated in "Experiments" section.

## Experiments

The decision tree model has been widely used for fraud detection^21–23^ as it produces easily interpretable decision rules whose logic is clearly laid out in the tree^11^. In addition, Baesens et al.^11^ suggest that careful feature engineering could improve the detection results of simple analytical techniques such as classification trees. To analyze the improvement of our feature construction rules, we input features belonging to different classifications to different machine learning models to examine their performance and feature importance. We obtained the transaction dataset from Bank L and the fraudulent accounts from the National Police Agency. The raw transaction dataset contains details for each transaction, like the account ID, transaction date, transaction amounts, transaction types, and account balances. If applicable, other information like textual messages, ATM IDs, E-bank, and telephony services are included. Bank L provides us with the definitions of 405 transaction types and maps each ATM ID to its owner branch. Here we used 30% of the account data for training, 30% for validation, and 40% for testing. The training data was re-sampled to ensure a 1 : 1 ratio between fraudulent and non-fraudulent accounts, whereas the ratio in the testing data followed the raw data ratio of 1 : 250. Precision (recall) rates denote the percentage of correctly identified fraudulent accounts to all accounts identified as fraudulent (real fraudulent accounts). There is a trade-off between these two rates; we examine the performance of each method using the F1 score. Our computer uses Intel(R) Core (TM) i7-10700 CPU with an RTX 3080 graphics card and 32GB DRAM.

Butaru et al.^12^ propose a method by which to evaluate the importance of features using the following three evaluation criteria to judge the importance of a feature *F*: (1) *weight*, denoting the number of times *F* appears in a tree, (2) *gain*, denoting the average gain of splits using *F*, and (3) *cover*, denoting the average coverage of splits which use *F*. Here coverage is defined as the number of samples affected by the split. We applied the atypical detection technique in "Features based on atypical detection techniques" section and the features proposed by other studies to the machine learning models to evaluate their importance. After removing the features listed in Table 2 with importance scores less than 0.1, the remaining features are listed in Table 3. The importance of the eight features proposed for our atypical feature extraction technique in Section "Features based on atypical detection techniques" annotated with “*” in Table 3 is significantly larger than 0 (no less than 0.1). Two of the five most important features, including the most important one, are proposed by our technique. This evidence attests the helpfulness of the proposed feature extraction rules for improving the performance of fraud detection models. The leave-one-out feature selection experiment^20^ in Table 4 shows that removing any of the proposed features generally reduces the precision rates, recall rates, and F1 scores significantly. Accuracy rates are slightly reduced due to strong data imbalance—the ratio between fraudulent and non-fraudulent accounts is 1 : 250. Adopting the RFM and FBADT features proposed in the literature degrades the performance of the machine learning model. We used these features to evaluate a variety of machine learning models; the experimental results are presented in Table 5. In addition, two of the top five most important features in Table 3 are not RFM or FBADT features, which also proves that using only these two categories of features degrades model performance.

**Table 3:** Feature importance.

Features	Feature categories	Importance	Rank
LT_Signature*	Others/non-fraudulent feature	1.80	1
*AVG_T_Interval*	Frequency/Basic statistical features	1.30	2
*Second_Most_Frequent_Act*	Frequency/Basic statistical features	1.07	3
*Credit_STDev*	Monetary/Basic statistical features	1.04	4
Saving_Gradient*	Others/Basic statistical features	0.97	5
*Most_Frequent_Act*	Frequency/Basic statistical features	0.78	6
*Debit_STDev*	Monetary/Basic statistical features	0.76	7
*24hr_Trade*	Recency/Basic statistical features	0.73	8
*Note_Not_NE*	Others/Basic statistical features	0.70	9
*BankID*	Others/Basic statistical features	0.69	10
*Least_Frequent_Act*	Frequency/Basic statistical features	0.68	11
*Magic_Number_Check*	Monetary/fraudulent feature	0.57	12
Weighted_Fraud_Coefficient*	Others/fraudulent feature	0.53	13
*LDT* >R*	Others/fraudulent feature	0.50	14
Withdrawal_After_Deposit*	Others/fraudulent feature	0.48	15
MFTAC*	others/fraudulent feature	0.46	16
LATM_Signature*	Others/non-fraudulent feature	0.27	17
HFW_Bank_of_ATM*	Others/fraudulent feature	0.20	18
*Suspicious_Branch*	Others/fraudulent feature	0.17	19

Features marked with “*” are unique features in our model and features listed as “Other” are not RFM or FBADT features.

(FBADT features do not appear here because the scores are too low and they are not included in Other).

**Table 4:** Magnitude of performance degradation caused by removing one of the features proposed in section "Features based on atypical detection techniques".

Removed feature	Accuracy attenuation	Precision attenuation	Recall attenuation	F1-score attenuation
LT_Signature	0.000363636	0.076502732	−0.030303030	0.014267447
Saving_Gradient	0.002727273	0.301282051	−0.060606061	0.134137758
Weighted_Fraud_Coefficient	0.001818182	0.246913580	0	0.118611547
LDT >R	0.000181818	0.026666667	0.030303030	0.029472443
Withdrawal_After_Deposit	0.000242424	0.034013605	0.045454545	0.042066146
MFTAC	0.000181818	0.032051282	0.015151515	0.021874547
LATM_Signature	0.000909091	0.145833333	0.136363636	0.142600090
HFW_Bank_Of_ATM	0.000303030	0.055555556	0.015151515	0.031196581

In the literature on feature selection, choosing effective feature classes is also an important issue. These results show that as the FBADT features proposed in the literature simultaneously consider multi-dimensional variables comprehensively, they perform worst in models that consider a single variable at a time. These results could be used as a reference for banks to improve their operating procedures and enhance security, and they may also serve as a clue when selecting features. From the experimental results, we observe that for most machine learning models, features in the monetary category perform best among the RFM and FBADT features. This result suggests that current research results on automatic feature engineering based on directly processing raw data and extracting frequency-related features may not be suitable for applications with real transaction datasets.

**Table 5:** Performance of RFM and FBADT features in various models.

Experimental results of the RFM features					Experimental results of the FBADT features				
Model	Accuracy (%)	Precision (%)	Recall (%)	F1-Score (%)	Model (%)	Accuracy (%)	Precision (%)	Recall (%)	F1-score (%)
XGBOOST with Recency	99.5	39.7	34.8	37.1	XGBOOST with FBADT	99.6	0.0	0.0	0.0
BayesPoint with Recency	8.3	0.4	100.0	0.9	BayesPoint with FBADT	1.5	0.4	97.0	0.8
RandomForest with Recency	93.0	3.7	65.2	6.9	RandomForest with FBADT	99.4	0.0	0.0	0.0
SVM with Recency	88.2	2.3	69.7	4.5	SVM with FBADT	36.4	0.4	68.2	0.8
NN with Recency	99.6	0.0	0.0	0.0	NN with FBADT	99.4	0.0	0.0	0.0
Logistic with Recency	95.9	0.2	1.5	0.3	Logistic with FBADT	99.6	0.0	0.0	0.0
LGBM with Recency	99.6	45.2	28.8	35.2	LGBM with FBADT	99.6	0.0	0.0	0.0
XGBOOST with Frequency	99.5	40.2	65.2	49.7	**Experimental results of using only the features proposed in past research**				
BayesPoint with Frequency	79.0	1.5	80.3	3.0	XGBOOST	99.6	0.0	0.0	0.0
RandomForest with Frequency	84.0	2.0	83.3	4.0	BayesPoint	1.5	0.4	97.0	0.8
SVM with Frequency	96.3	7.5	72.7	13.5	RandomForest	98.8	9.6	22.7	13.5
NN with Frequency	96.8	8.2	69.7	14.7	SVM	96.7	8.9	77.3	16.0
Logistic with Frequency	95.1	6.1	77.3	11.3	NN	89.8	0.2	4.5	0.4
LGBM with Frequency	99.6	45.7	48.5	47.1	Logistic	99.6	0.0	0.0	0.0
XGBOOST with Monetary	99.8	82.6	57.6	67.9	LGBM	99.6	0.0	0.0	0.0
BayesPoint with Monetary	14.8	0.5	100.0	0.9					
RandomForest with Monetary	96.2	8.1	81.8	14.8					
SVM with Monetary	97.3	10.3	75.8	18.1					
NN with Monetary	41.0	0.5	78.8	1.1					
Logistic with Monetary	88.7	0.7	18.2	1.3					
LGBM with Monetary	99.7	63.3	57.6	60.3					

In addition to the above-mentioned features based on statistical data, Xie et al.^7^ adopt a similar approach to ours involving features based on financial expertise, and establish a machine learning model to detect credit card fraud. However, their features are almost all RFM features, unlike ours which are specifically designed for normal transactions or money laundering. For comparison, we list the features that are suitable for our dataset in Table 6 and evaluate them with various machine learning models. We find that machine learning models perform poorly if only their proposed features are used. Hence, we experiment with their proposed features together with the features used in our model. That is, we add the features proposed by Xie et al. to our model. We present the results of these experiments in Table 7: the results indicate that when using all the features, the model performs better than when using only the features proposed by Xie et al. However, this result is outperformed by our model presented below, which proves that with these models, more features does not necessarily translate to better performance. From Table 8, we observe that adding features that identify fraudsters or non-fraudsters to the RFM features improves the performance of most models, which may explain why our proposed features outperform those proposed by Xie et al.

**Table 6:** Features proposed by Xie et al.

Sensitive single amount count	
Sensitive daily total amount count	
Sensitive test amount count	
Large amount count	
Untrusted frequent trade count	
Big one-time deal count	
Amount over month	
Average daily over month	
Average over 2 months	
Amount Transaction object over month	
Number Transaction object over month	
Amount Transaction object over 2 months	
Max Amount same day	
Max Number same day

**Table 7:** Performance of features proposed by Xie et al. (X) and our proposed features (O).

Model	Accuracy	Precision	Recall	F1-Score
XGBOOST with X	99.8%	78.4%	60.6%	68.4%
BayesPoint with X	8.4%	0.4%	100.0%	0.9%
RandomForest with X	92.3%	4.5%	89.4%	8.5%
SVM with X	97.2%	10.3%	78.8%	18.2%
NN with X	28.0%	0.5%	98.5%	1.1%
Logistic with X	93.8%	3.2%	50.0%	6.1%
LGBM with X	99.8%	79.2%	57.6%	66.7%
XGBOOST with X+O	99.8%	91.3%	63.6%	75.0%
BayesPoint with X+O	11.5%	0.4%	100.0%	0.9%
RandomForest with X+O	97.0%	10.4%	86.4%	18.6%
SVM with X+O	99.6%	47.9%	69.7%	56.8%
NN with X+O	99.5%	0.0%	0.0%	0.0%
Logistic with X+O	93.8%	1.7%	25.8%	3.2%
LGBM with X+O	99.7%	72.5%	56.1%	63.2%
Rule-based	0.993	0.400	0.056	0.098

We also compare the non-fraudulent features and fraudulent features shown in Table 1. Due to the poor performance of the FBADT features, these experiments do not use the FBADT features.

**Table 8:** Performance using statistics-based features (SB), non-fraud features (L), and fraud features (F).

model	Accuracy	Precision	Recall	F1-Score
XGBoost with SB	0.998	0.872	0.515	0.648
Logistic Regression with SB	0.984	0.171	0.803	0.282
Bayes Point Machine with SB	0.988	0.205	0.682	0.315
Decision Forest with SB	0.997	0.681	0.485	0.566
Support Vector Machine with SB	0.987	0.193	0.697	0.303
Neural Network with SB	0.997	0.581	0.652	0.614
Decision Jungle with SB	0.975	0.114	0.758	0.198
XGBoost with SB+L	0.998	0.902	0.561	0.692
Logistic Regression with SB+L	0.986	0.2	0.833	0.323
Bayes Point Machine with SB+L	0.99	0.24	0.667	0.353
Decision Forest with SB+L	0.997	0.769	0.455	0.571
Support Vector Machine with SB+L	0.988	0.209	0.727	0.324
Neural Network with SB+L	0.997	0.61	0.712	0.657
Decision Jungle with SB+L	0.963	0.075	0.727	0.137
XGBoost with SB+F	0.998	0.941	0.485	0.64
Logistic Regression with SB+F	0.989	0.24	0.818	0.371
Bayes Point Machine with SB+F	0.992	0.3	0.773	0.432
Decision Forest with SB+F	0.998	0.917	0.5	0.647
Support Vector Machine with SB+F	0.992	0.297	0.788	0.432
Neural Network with SB+F	0.997	0.648	0.697	0.672
Decision Jungle with SB+F	0.97	0.107	0.879	0.19
XGBoost SB+L+F **(Our model)**	0.998	0.87	0.712	0.783
Logistic Regression SB+L+F	0.99	0.261	0.864	0.401
Bayes Point Machine SB+L+F	0.994	0.368	0.803	0.505
Decision Forest SB+L+F	0.998	0.85	0.515	0.642
Support Vector Machine SB+L+F	0.992	0.294	0.803	0.431
Neural Network SB+L+F	0.997	0.637	0.773	0.699
Decision Jungle SB+L+F	0.971	0.098	0.773	0.174

The proposed model uses statistics-based features (SB), non-fraud features (L), and fraud features (F) on XGBoost.

Table 8 presents the performance of the XGBoost, Bayes point machines, decision forests, support vector machines (SVMs), neural networks (NNs), decision jungles, and logistic regression using the features introduced in Table 1. The results show that when only statistics-based (SB) features are used, all machine learning models perform poorly. It is necessary to add features that differentiate normal transactions from money laundering. Compared with various previous attempts, using our selected features on XGBoost yields the best performance.

Although Tai and Kan^8^ report a strong trade-off between precision and recall rates, our XGBoost model produces the best F1 score of 78.3%, which is more than eight times that of the rule-based method (9.756%). Nevertheless, the Bayes point machine is indicated if the ability to detect fraudulent accounts is more important than high precision. Thus we confirm the argument from Baesens et al.,^11^ that is, that careful feature engineering could yield good detection results even when using analytical techniques as simple as classification trees.

## Conclusion and future research

Detecting frauds with machine learning has become a hot topic due to the limit of traditional rule-based fraud detection mechanisms. Much literature uses features based on RFM (i.e., recency, frequency, monetary) and FBADT (i.e., anomaly detection techniques) to train machine learning models, like XGBoosts, Bayesian Point Machines, decision forests, SVMs, NNs, and Logistic Regression studied in this paper. However, detecting frauds with the aforementioned statistical-based features can be unstable, especially including FBADT features causes some models to perform very poorly. Hence, this paper addresses this problem by systematically generating features from three aspects (basic statistics, characteristics of fraudulent actors, and characteristics of non-fraudulent actors) through monitoring transactions and AML guidelines.

Specifically, the second aspect captures fraudulent patterns according to financial expertise and cases provided by judicial authorities. The third aspect focuses on normal transaction patterns seldom involved in fraudulent acts. Experimental results prove the superiority of our proposed features, especially those that characterize normal and fraudulent accounts. The F1-score produced by training XGBoost with all our proposed features outperforms other machine learning models using RFM and FBADT features. It is more than eight times higher than the rule-based method adopted by our partner bank. According to financial ranking criteria, many of the features we propose are important. The explainability of the XGBoost model also allows us to target key features that describe fraudsters’ profiles, helping banks improve the transaction processes and system security.

Generating effective and interpretable features to improve fraudulent detection performance and explainability is essential in the financial industry and regulators. Recently, we obtained more detailed real transaction data, like the bank customers’ information (e.g., occupations) and (blood and legal) relations among customers from our extended industrial cooperation project. Creating feasible features to capture meaningful characteristics of (non)-fraudulent actors embedded in complex real transaction data could be an exciting but challenging future work. In addition to constructing features based on statistical methods and financial expertise as proposed in this paper, we plan to use the feature synthesis approach and other more complex machine learning methods to improve the fraudulent detection performance. However, even though adopting the aforementioned “black-box” models might improve fraud detection ability, persuading a financial authority to approve banks to adopt these complex models could be difficult due to interpretability concern. Besides, fraudsters may attract innocent people to commit fraud; the behaviors of these people’s accounts may be normal but involved in fraudulent activities in the future. Thus, conducting risk assessments for each customer according to behaviors changes becomes critical. Capturing time-varying patterns and spotting normal customers likely to become fraudsters is another challenging future work.

## Data Availability

The data that support the findings of this study are available from Metaedge Corporation Taiwan but restrictions apply to the availability of these data, which were used under license for the current study, and so are not publicly available. Data are however available from the authors upon reasonable request and with permission of Metaedge Corporation Taiwan.
